# The Plague in India

**Published:** 1898-02-26

**Authors:** 


### The Hospital

A Journal of

The Medical Sciences and Hospital Administration.

Vol. XXIII.—No. 596]. Edited by Sir Henry Burdett, K.C.B., and Solomon C. Smith, M.D., M.R.C.P. [Feb. 26, 1898.

### THE PLAGUE IN INDIA.

The very important and interesting paper on Plague in India, which was read by Mr. H. M. Birdwood before the Indian Section of the Society of Arts, at the Imperial Institute, on the 17th instant, was the first official and trustworthy account of the outbreak which had reached this country. The newspaper reports had all been more or less fragmentary, or had taken account only of such phenomena as most impressed the observers who recorded them; but Mr. Birdwood, himself a member of the government of Bombay, and also of a family which for many years has been identified with the prosperity of the "Royal and Dower-Royal City," was from the first a spectator of the horrors which he recorded, as well as of the heroic, or, as the late Sir Henry Havelock-Allan called them, the "superhuman" efforts which were made for their mitigation. The epidemiology of India, as was first pointed out by the late Mr. Netten Radcliffe, has always been of so complicated a character as to baffle all but the most studious and the most pains-taking inquirers, if only on account of the number of channels for the communication of disease which have existed on every side. We are somewhat disposed to think that the increased precision of knowledge which has been afforded by bacteriology has not been entirely without drawbacks, in the tendency which it has had to restrict investigation to the mere presence of an essential bacillus, independently of the conditions which either promote or hinder its multiplication and its activity. Sir John Simon, many years ago, when speaking with the intuition of genius of the causation of cholera, said "excrement polluted earth, excrement polluted water, excrement polluted air, these are for us the causes of cholera;" and his worthy successor, Sir Richard Thorne, in the debate at the Imperial Institute, said that what India now wanted was not so much science as practice. All who know the country, or at least all who know the ways and habits of the natives, know also that their very religions are insanitary, and that, even in a greater degree than in Europe in the middle ages, piety and filth are united together by bonds that are practically indissoluble. The dangerous character of the combination is well known to increase in a geometrical ratio with increase of population upon space; and conditions, which might be harmless in a loosely populated city, become deadly where the inhabitants are crowded together. The lesson taught by plague in England, while it still prevailed here, is that it does not speedily loosen its hold upon any city in which it finds a home, but that its tendency is to return, year after year, until either the susceptible material is exhausted, or until such changes have been wrought as interfere with the security of its tenure. As regards Bombay, the very remarkable evidence of Dr. Cleghorn, given at the International Sanitary Congress of Venice, and repeated by Sir Richard Thorne on the 17th, shows the existence of centres of disease from which it is impossible that the infective material should be soon or completely eradicated; and the details, which Sir Richard spared his hearers, but which were set forth with grim precision in the original paper, were quite sufficient to justify the statement that no such hotbeds of pestilence had ever before been permitted to exist in a civilised country, or under the government of Englishmen. Wherever plague has prevailed, there has been the same ghastly tale of filth, domestic and personal. The habits of the lower classes of Chinese are only too well known; and Colvill, in his account of the plague in Persian Kurdistan, writes "that whatever is most afflicting in poverty, whatever is most revolting in filthiness, is accumulated, as if designedly, around these infected dens, in which live, or rather vegetate, from fifty to sixty men, women, and children." There is great reason to hope, from Mr. Birdwood's narrative, as well as from the possibly less authentic stories of other writers, that the labours and the courage of Europeans, in facing and in endeavouring to subdue the epidemic, are beginning to be appreciated at their true worth by the natives of India; and, if this be so, there can be no doubt that advantage should be taken of the fact in order to introduce into the country, and to push forward with such despatch as may be possible, the ordinary sanitary work which we regard as having reference to the maintenance of health and of vitality in general, with no particular reference to any single form of disease. There has been too much fear of the "prejudices of the natives" in the matter of dirt; and the time has come at which its universal prevalence can no longer be safely disregarded. The security given by the British dominion has led to an enormous increase of population in India; and it is admitted by all who understand the subject that overcrowding almost indefinitely increases the dangers of dirt.

